# Supplementary material for: Capturing relativistic wakefield structures in plasmas using ultrashort high-energy electrons as a probe
Source: Sci Rep. 2016 Jul 11;6:29485. doi: 10.1038/srep29485 (PMC4939525; doi:10.1038/srep29485)
Supplement: Supplementary Information [file srep29485-s1.pdf]

Capturing relativistic wakefield structures in plasmas using ultrashort high-energy electrons as a probe

C. J. Zhang[1,2], J. F. Hua[1], X. L. Xu[3], F. Li[1,2], C.-H. Pai[1], Y. Wan[1,2], Y. P. Wu[1], Y. Q. Gu[2], W. B. Mori[3], C. Joshi[3], and W. Lu[1,*]

[1]Tsinghua University, Beijing 100084, China
[2]Laser Fusion Research Center, Mianyang, Sichuan 621900, China
[3]University of California Los Angeles, Los Angeles, California 90095, USA
[*]weilu@tsinghua.edu.cn

ABSTRACT

A new method capable of capturing coherent electric field structures propagating at nearly the speed of light in plasma with a time resolution as small as a few femtoseconds is proposed. This method uses a few femtoseconds long relativistic electron bunch to probe the wake produced in a plasma by an intense laser pulse or an ultra-short relativistic charged particle beam. As the probe bunch traverses the wake, its momentum is modulated by the electric field of the wake, leading to a density variation of the probe after free-space propagation. This variation of probe density produces a snapshot of the wake that can directly give many useful information of the wake structure and its evolution. Furthermore, this snapshot allows detailed mapping of the longitudinal and transverse components of the wakefield. We develop a theoretical model for field reconstruction and verify it using 3-dimensional particle-in-cell (PIC) simulations. This model can accurately reconstruct the wakefield structure in the linear regime, and it can also qualitatively map the major features of nonlinear wakes. The capturing of the injection in a nonlinear wake is demonstrated through 3D PIC simulations as an example of the application of this new method.

Supplemetary materials

Effect of magnetic field

In our theoretical model, the magnetic field in the wake is neglected when calculating the momentum modulation caused by wakefield in Eq. (1). It turns out that the momentum change caused by magnetic field is rather small comparing with the contribution of the electric field. The integrated field $I_E = \int E_z dx$ and $I_B = \int B_\theta dx$ are shown in Fig. S1 (a) and (b) respectively. The integrated fields are proportional to the momentum modulation. Figure S1 (c) shows the axial lineout for I_E (the blue line) and I_B (the red line). The large peak of the magnetic field at $z \approx 900$ µm is generated by the driver. The contribution from the magnetic field is less than 4% for this linear case. In the highly nonlinear regime, the magnetic field is stronger. However, the contribution from the electric field is still much larger than that from the magnetic field, as shown in Fig. S1 (d) and (e) respectively. The axial lineouts for I_E (blue line) and I_B (red line) are shown in Fig. S1 (f). For this highly nonlinear case, the contribution from the magnetic field is less than 10% of that from the electric field.

Reconstruction of wake driven by a non-Gaussian driver

Figure S2 shows the reconstruction results of a wake generated by an electron beam driver with a super-Gaussian transverse profile. The simulation parameters are the same as those in Fig. 3, except for the transverse density profile is modified to $n_b(r) = \exp(-r^4/2\sigma^4)$, where $\sigma = 25.5$ µm. Figure S2 (a) shows the probe density perturbation on the virtual screen, which is placed 10 mm behind the plasma. Figure S2 (b) and (c) show the reconstructed E_z and E_r field components respectively. The axial lineout is shown in Fig. S2 (d), where the red dashed line represents the reconstructed field, which agrees very well with the simulated field (the blue solid line). Figure S2 (e) and (f) show the radial lineout of E_z and E_r at the positions indicated by the black dashed line in (b) and (c) respectively. Although the transverse distribution of the density perturbation as well as the E_z field have no longer Gaussian forms, the reconstruction results still agree very well with the simulated fields. This further demonstrates the usefulness of the proposed method.

Derivation of K_τ, K_E and K_ε

For a probe with length of τ (defined as the full width at half maxima of the current profile, in unit of λ_p), the density perturbation will be $\delta n/n_0 = K_\tau(\delta n/n_0)_0$, where $(\delta n/n_0)_0$ is the density perturbation for the ideal probe. The axial field of a linear wake is

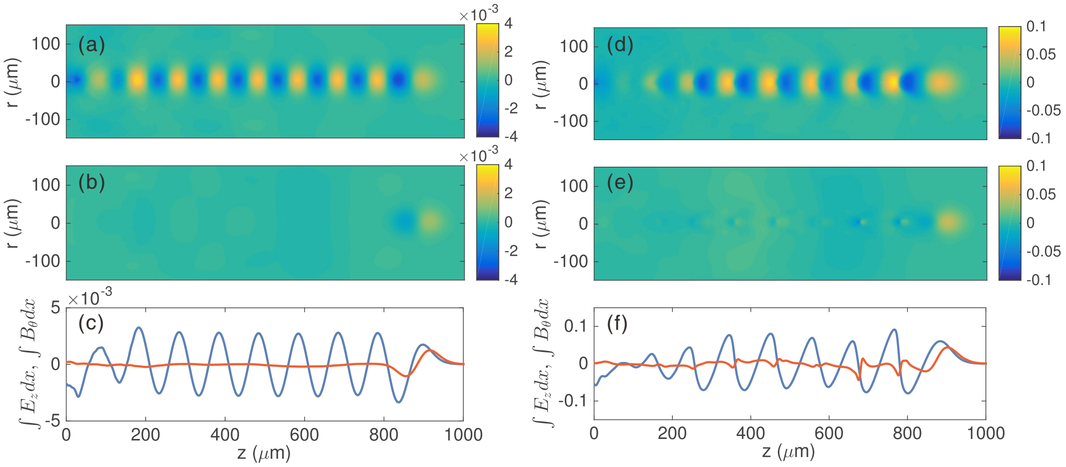

Figure S 1. Effect of the magnetic field. (a) Momentum modulation contributed from the electric field $I_E = \int E_z dx$. (b) Momentum modulation contributed from the magnetic field $I_B = \int B_\theta dx$. (c) Axial lineout of I_E (the blue line) and I_B (the red line). (a)-(c) show the results for a linear wakefield. (d)-(f) show the similar results for a nonlinear wakefield.

$E_z \sim \exp(-r^2/2\sigma_E^2)\sin(k_p z - \omega_p t)$, where σ_E is the width of the wakefield. We can drop the exponential term because it doesn't depend on time. Assume that the current profile of the probe is $I(t)$, then the integral of the field felt by different parts of the probe will be $\int \sin(k_p z - \omega_p t) I(t) dt / \int I(t) dt$. If the probe has a flat-top current profile, i.e., $I(t) = 1$ for $0 < t < \tau_0$, the equation can be rewritten as $\int_0^{\tau_0} \sin(k_p z - \omega_p t) dt/\tau_0 = \frac{\sin(\omega_p \tau_0/2)}{\omega_p \tau_0/2}\sin(k_p z - \omega_p \tau_0/2)$. Thus the correction factor will be $K_\tau = \frac{\sin(\omega_p \tau_0/2)}{\omega_p \tau_0/2}$ and can be rewritten as $K_\tau = |\frac{\sin(\pi\tau)}{\pi\tau}|$, where $\tau = c\tau_0/\lambda_p$ is the probe length and $|\cdot|$ means to take the absolute value to ensure the correction factor to be positive for any probe length. For a probe with $\sin^2$ current profile, $I(t) = \sin^2(\pi t/2\tau_0)$, the correction factor can be derived in a similar way and the result is $K_\tau = |(\frac{1}{2\pi\tau} - \frac{1}{2\pi(2\tau-1)} - \frac{1}{2\pi(2\tau+1)})\sin(2\pi\tau)|$, where $\tau = c\tau_0/\lambda_p$ is applied. For a probe having Gaussian current profile, the correction factor is $K_\tau = \exp(-(k_p\sigma_\tau)^2/2)$, where $\sigma_\tau = \tau\lambda_p/2\sqrt{2\ln 2}$.

For a probe with relative energy spread of $x = \Delta E/E_0$, the density perturbation will be $K_E(\delta n/n_0)_0$. Here E_0 is the central energy and ΔE is the FWHM energy spread, as shown in the inset in Fig. 5 (b). The density perturbation of an ideal probe is $\delta n/n_0 \propto \nabla \cdot \frac{\delta p}{p_0}$. Assume that the energy spectrum of the probe has a uniform distribution, $n(p) = \int n(p)dp/(xp_0)$, where p_0 is the central momentum and the momentum varies from $p_0 - xp_0/2$ to $p_0 + xp_0/2$, then the density perturbation will be

$$\frac{\delta n}{n_0} = \frac{\int \delta n(p) dp}{\int n(p) dp} = \nabla \cdot \int \frac{1}{xp_0}\frac{\delta p}{p} dp = \int_{1-x/2}^{1+x/2} \frac{1}{x}\frac{1}{p} dp \nabla \cdot \frac{\delta p}{p_0} = \frac{1}{x}\ln\left(\frac{2+x}{2-x}\right)\left(\frac{\delta n}{n_0}\right)_0$$

Thus the correction factor is $K_E = x^{-1}\ln(\frac{2+x}{2-x})$. For a probe with $\sin^2$ energy spectrum, a similar formula can also be derived, $K_E = x^{-1}\int_{1-x}^{1+x}\cos^2(\frac{\pi(\alpha-1)}{2x})\frac{1}{\alpha}d\alpha$, where $\alpha = p/p_0$.

For a given beam spot size σ_x, the emittance ε is proportional to the slice momentum spread in the phase space ($\varepsilon \propto \sigma_x \sigma_{p_x}/p_0$), as shown by the inset in Fig. 5 (c). The slices of the probe marked by 1 and 2 in the inset in Fig. 5 (c) have different initial transverse momentum p_{x1} and p_{x2}, which will introduce a transverse displacement between them after free-space propagation. Assume that the density perturbation of each slice caused by the wake along the wake propagation direction is $\sin(k_p x)$, and the displacement between two slices is $d \propto L\delta p_x/p_0$, then the total density perturbation will be $2\cos(k_p d/2)\sin(k_p x + k_p d/2)$, which has a smaller amplitude than $2\sin(k_p x)$ for the ideal probe where $p_{x1} = p_{x2}$. This means that the density perturbation will decrease as the emittance increases. Assume that the wake is linear and the slice spread of the transverse momentum p_x is σ_{p_x}, which doesn't change for different x for simplicity, then the correction factor can be derived as $K_\varepsilon = e^{-(k_p\sigma_\theta L)^2/2}$ in a similar way as the derivation of effect of the probe length, where $\sigma_\theta = \sigma_{p_x}/p_0$ and L is the drift distance.

The drift distance

The distance between the screen and the plasma should be kept as small as possible to avoid crossing of the trajectories of electrons at different initial positions. For typical electron probes generated from a laser-plasma wakefield accelerator the distance between the screen and the wake should be smaller than $L_m \approx M\gamma\lambda_p/10$, where γ is the Lorentz factor for the probe, λ_p is the plasma wavelength and M is the geometrical magnification factor. This formula is obtained by assuming that

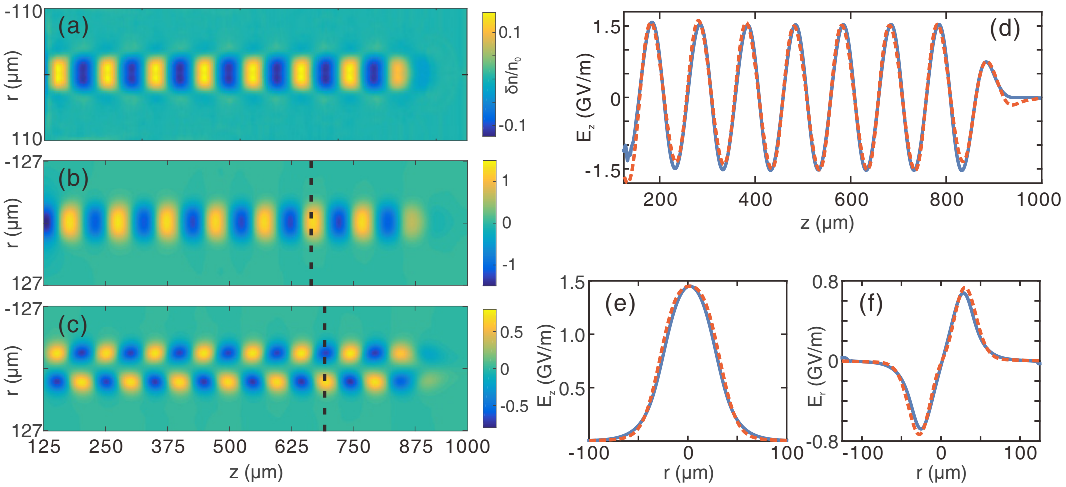

Figure S 2. (a) Probe density perturbation as recorded on a virtual screen. (b) and (c) are the reconstructed E_z and E_r field components, respectively. (d) On-axis ($r = 0$) lineout of reconstructed (red dashed line) and simulated (blue solid line) E_z. (e) and (f) show the transverse lineout of E_z and E_r at the positions indicated by the black dashed line in (b) and (c), respectively. The blue solid lines are for the simulated fields, while the red dashed lines are for the reconstructed fields.

the amplitude of the wakefield is equal to the cold plasma wave breaking limit $E_0 = m_e c\omega_p/e$ and by limiting the density perturbation $\delta n/n_0 \leq 1$. L_m is a lower limit because the actual amplitude of the electric field in a linear wake is usually much smaller than the wave breaking limit. The distance L varies from a few mm to tens of cm, depending on the probe energy and plasma parameters.
